# Sex, Age, and Bacteria: How the Intestinal Microbiota Is Modulated in a Protandrous Hermaphrodite Fish

**DOI:** 10.3389/fmicb.2019.02512

**Published:** 2019-10-31

**Authors:** M. Carla Piazzon, Fernando Naya-Català, Paula Simó-Mirabet, Amparo Picard-Sánchez, Francisco J. Roig, Josep A. Calduch-Giner, Ariadna Sitjà-Bobadilla, Jaume Pérez-Sánchez

**Affiliations:** ^1^Fish Pathology Group, Institute of Aquaculture Torre de la Sal (CSIC), Castellón, Spain; ^2^Nutrigenomics and Fish Growth Endocrinology Group, Institute of Aquaculture Torre de la Sal (CSIC), Castellón, Spain; ^3^Biotechvana S.L., Valencia, Spain; ^4^Instituto de Medicina Genomica, S.L., Valencia, Spain

**Keywords:** gilthead sea bream, intestinal microbiota, age, sex, intestinal health

## Abstract

Intestinal microbiota is key for many host functions, such as digestion, nutrient metabolism, disease resistance, and immune function. With the growth of the aquaculture industry, there has been a growing interest in the manipulation of fish gut microbiota to improve welfare and nutrition. Intestinal microbiota varies with many factors, including host species, genetics, developmental stage, diet, environment, and sex. The aim of this study was to compare the intestinal microbiota of adult gilthead sea bream (*Sparus aurata*) from three groups of age and sex (1-year-old males and 2- and 4-year-old females) maintained under the same conditions and fed exactly the same diet. Microbiota diversity and richness did not differ among groups. However, bacterial composition did, highlighting the presence of *Photobacterium* and *Vibrio* starting at 2 years of age (females) and a higher presence of *Staphylococcus* and *Corynebacterium* in 1-year-old males. The core microbiota was defined by 14 Operational Taxonomic Units (OTUs) and the groups that showed more OTUs in common were 2- and 4-year-old females. Discriminant analyses showed a clear separation by sex and age, with bacteria belonging to the phyla Firmicutes, Proteobacteria and Actinobacteria driving the separation. Pathway analysis performed with the inferred metagenome showed significant differences between 1-year-old males and 4-year-old females, with an increase in infection-related pathways, nitrotoluene degradation and sphingolipid metabolism, and a significant decrease in carbohydrate metabolism pathways with age. These results show, for the first time, how intestinal microbiota is modulated in adult gilthead sea bream and highlight the importance of reporting age and sex variables in these type of studies in fish.

## Introduction

The consumption of fish is continuously promoted for its multiple health benefits (Hamed et al., 2015; Steffens, 2016). This increase in demand together with the stagnation of fisheries, mainly due to overexploitation of wild stocks, have converted aquaculture in the fastest-growing animal food production sector in the world with an increasing contribution to global food supply (Delgado et al., 2003; FAO, 2016). Gilthead sea bream (*Sparus aurata* L.) is the main cultured fish in the Mediterranean basin and the third most important produced species in Europe. This fish is a sequential protandrous hermaphrodite. It matures first as male and in the following cycles – depending on social factors, growth, and diet – female reproductive organs develop (Mylonas et al., 2011; Simó-Mirabet et al., 2018). Just like in higher vertebrates, the intestinal microbiota of fish is key for many host functions, such as digestion, nutrient metabolism and absorption, disease resistance, immune function, and tissue development (Rawls et al., 2004). With the growth of the aquaculture industry, there has been a growing interest in the modulation of fish gut microbiota to improve welfare and nutrition (Egerton et al., 2018). However, a long road lies ahead to establish the baseline parameters to guide this manipulation.

Since the advent of new sequencing technologies, many studies have been conducted on fish intestinal microbiota. These studies show that microbial communities display great intra- and inter-specific variations. Natural factors that affect this diversity include trophic level, diet, season, habitat, captive-status, age, sex, and genetics (some examples of the multitude of studies assessing this are Bano et al., 2007; Dhanasiri et al., 2011; Hovda et al., 2012; Clements et al., 2014; Cordero et al., 2015; Stephens et al., 2016; Li et al., 2017; Piazzon et al., 2017; Jones et al., 2018; Kokou et al., 2018; Navarro-Barrón et al., 2019; Zhang et al., 2019). Intestinal microbiota also changes within the same individual in different parts of the intestine due to their physiological differences (Ringø et al., 2006; Jones et al., 2018). Moreover, the transient microbiota in intestinal contents (allochthonous) of fish and mammals differs from the adherent (autochthonous) communities (Kim et al., 2007; Durban et al., 2011). Lastly, the experimental setup (rearing conditions, recirculation vs. open flow systems, temperature, and photoperiod), DNA extractions techniques and methodologies for assessing the communities (454 pyrosequencing, Illumina sequencing, PCR-DGGE) have a large impact in the results of this type of studies (Piazzon et al., 2017). All these sources of variation make the establishment of a baseline microbiota difficult and highlight the importance for all the studies to report all details of the experimental setup. However, many studies suggest that the autochthonous microbiota colonizing the intestinal mucosal surface of a given species, which makes up the core microbial community and directly interact with the host physiology, can persist in spite of changing factors (Roeselers et al., 2011; Egerton et al., 2018).

The microbiota of vertebrate intestines consists of complex communities of microbes including viruses, protozoa, yeast, archaea, and bacteria (Merrifield and Rodiles, 2015). The aim of this study was to characterize and compare the autochthonous intestinal bacterial microbiota of adult gilthead sea bream (*S. aurata*) from three different groups of age reared in the same conditions. We also studied the potential significance of the changes in the biology and welfare of the reared animals. The overall objective of this line of work is to establish reference points in the bacterial composition of gilthead sea bream in order to better understand and improve the welfare and growth performance of this species in aquaculture.

## Materials and Methods

### Animals and Samplings

One-, 2- and 4-year-old gilthead sea bream (*S. aurata* L.) of Atlantic origin were kept from early life stages under the same rearing conditions and feeding regime with commercial pellets (BIOMAR: INTRO PLUS MT 1.9 mm, EFICO YM 854 3–4.5 mm) at the indoor experimental facilities of the Institute of Aquaculture Torre de la Sal (IATS-CSIC, Spain). Fish were kept in an open-flow system and fed *ad libitum* once-twice per day, 3–6 days per week, depending on the season and fish size. To minimize individual variability, all fish were fed the same batch of finishing pellets (EFYCO YM 854 6.5 mm) 2 months before tissue sampling. Oxygen content of outlet water remained higher than 75% saturation, and day length and water temperature followed natural changes at IATS latitude (40° 5′ N; 0° 10′ E).

After a 2-day fasting period, 10 fish per age group, were sacrificed in summer (July 2018, water temperature 22–24°C) under reproductive quiescence by overexposure to the anesthetic 3-aminobezoic acid ethyl ester (MS-222, 0.1 g/l) and biometric measurements were taken. Intestines were dissected and the anterior portion was cut out, opened and gently washed with sterile PBS to remove non-adherent bacteria. The anterior portion of the intestine was chosen due to its important role in nutrient absorption and metabolism. Autochthonous bacteria were targeted because these are the organisms colonizing the mucosal surface and having a direct impact on the physiology of the animal, whereas allochthonous bacteria cannot colonize these habitats under normal conditions (Hao and Lee, 2004). Intestinal mucus was scrapped off with the blunt edge of a sterile scalpel and collected in sterile 1.5 ml tubes. DNA extraction was performed immediately after the sampling, thus, samples were kept on ice for a maximum of 2 h. All fish were sampled within 3 days to avoid differences due to changes in bacterial composition of the water.

### Ethics Statement

All procedures were approved by the Ethics and Animal Welfare Committee of IATS and CSIC. They were carried out in a registered installation facility (code ES120330001055) in accordance with the principles published in the European Animal Directive (2010/63/EU) and Spanish laws (Royal Decree RD53/2013) for the protection of animals used in scientific experiments.

### DNA Extraction

Up to 200 μl of mucus samples were treated with 250 μg/ml of lysozyme (Sigma) for 15 min at 37°C. The DNA was extracted with the High Pure PCR Template Preparation Kit (Roche) following the manufacturer’s instructions. DNA concentration and quality was checked by Nanodrop 2000c (Thermo Scientific) and agarose gel electrophoresis (1% w/v in Tris-EDTA buffer). DNA was stored at −20°C until used for sequencing.

### Illumina MiSeq Sequencing of 16S rRNA Amplicons

Sequencing of the V3–V4 region of the 16S rRNA gene (reference nucleotide interval, 341–805 nt) was performed using the Illumina MiSeq system at the Unidad de Genómica del Parque Científico de Madrid (FPCM). DNA concentration was measured with Picogreen (Thermo Fisher) and 3 ng of each sample were used for the first PCR round using the Q5^®^ hot Start High-Fidelity DNA Polymerase (New England Biolabs) and 100 nM of primers, in a final reaction volume of 25 μl. The primers used were CS1-341F (5′-ACACTGACGACATGGTTCTACACCTACGGGNGGCWGCA G-3′) and CS2-805R (5′- TACGGTAGCAGAGACTTGGTCTGA CTACHVGGGTATCTAATCC-3′). Cycling conditions were 98°C 30 s; followed by 26 cycles of 98°C 10 s, 50°C 20 s, and 72°C 20 s; with a final elongation step at 72°C 2 min. A second PCR round was conducted using 1 μl of the first PCR reaction, the same enzyme and 400 nM of primers (F: 5′-AATGATACGGCGACCACCGAGATCTACACTGACGACATG GTTCTACA-3′/R: 5′-CAAGCAGAAGACGGCATACGAGAT-[10 nucleotides barcode]-TACGGTAGCAGAGACTTGGTCT-3′, Fluidigm, Access Array Barcode Library for Illumina Sequencers) in a 20 μl reaction volume. Cycling conditions were the same as in the first round with the exception that only 12 cycles were used.

PCR products were checked and quantified with Bioanalyzer (Agilent) and purified with AMPure Beads (Beckman Coulter). The amplicon pool was quantified by *q*PCR using Kapa-SYBR FAST *q*PCR kit for LightCycler480 and a reference library from the Unidad de Genómica FPCM. An equimolar pool of amplicons was sequenced in Illumina MiSeq (2 × 300 paired-end run) following the manufacturer’s instructions and using the MiSeq Reagent Kit v3 600 Cycles. Raw sequenced data obtained was lodged in the Sequence Read Archive (SRA) under the Bioproject accession number PRJNA554554 (BioSample accession numbers: SAMN12273608-37).

### Bioinformatic Analysis

Raw forward and reverse sequences were quality-filtered using FastQC^1^. The reads were then pre-processed using Prinseq (Schmieder and Edwards, 2011). When needed, terminal N bases were trimmed in both ends and sequences with >5% of total N bases were discarded. All reads that were <150 bp long, had a Phred quality score <28 in both of the sequence ends and had a Phred average quality score <26 were excluded. Forward and reverse pre-processed reads were merged using the fastq-join-based script join_paired_ends.py, from the QIIME package (Caporaso et al., 2010).

Taxonomy was assigned using the Ribosomal Database Project (RDP) release 11 (combination of Archaea+Bacteria) as reference database (Cole et al., 2014). Reads were aligned with a custom-made pipeline using VSEARCH (Altschul et al., 1990; Sambles et al., 2017). Alignment was performed stablishing high stringency filters (≥90% sequence identity, ≥90% query coverage). Taxonomic assignation results were filtered and data was summarized in an Operational Taxonomic Units (OTUs) table. The aforementioned protocols for data filtering and taxonomic assignation were managed using the GPRO suite software (Futami et al., 2011). Sample depths were normalized by total sum scaling and then made proportional to the total sequencing depth using the formula (count×total counts in all samples)/total sample counts, following the recommendations described elsewhere (McKnight et al., 2019).

### Statistical Analysis

Rarefaction curves were obtained by plotting the number of observed taxonomic assignations against the number of sequences. Species richness estimates and alpha diversity indexes were calculated using the R package Phyloseq (McMurdie and Holmes, 2013). Differences among age groups in biometric data were assessed by one-way ANOVA (Holm–Sidak post-test). Differences in species richness and diversity indexes were determined by Kruskal–Wallis test (Dunn’s post-test). Comparisons of phyla abundance among age groups were performed by two-way ANOVA that detected no interactions and significant differences at the level of phylum among groups. Thus, the different abundance of each phylum among groups was compared by Kruskal–Wallis test (Dunn’s post-test). Differences were considered significant when *P*-value < 0.05.

A hierarchical representation of the taxonomic assignments hierarchies of microbial communities was performed using Krona (Ondov et al., 2011). Beta-diversity across age groups was tested with permutational multivariate analysis of variance (PERMANOVA) using the R package Vegan. The distance matrix was constructed with the *betadiver* function (method = “z”) and then the non-parametric method *adonis* (1000 random permutations) was applied. To study the separation among experimental groups, partial least-squares discriminant analysis (PLS-DA) was performed using EZinfo v3.0 (Umetrics, Umeå, Sweden). Hotelling’s *T*^2^ statistic was calculated by the multivariate software package and points above 95% confidence limit for *T*^2^ were considered as outliers and discarded. The quality of the PLS-DA model was evaluated by the parameters R2Y (cum) and Q2 (cum), which indicate the fit and prediction ability, respectively. To discard the possibility of over-fitting of the supervised model, a validation test consisting in 999 random permutations was performed using SIMCA-P+v11.0 (Umetrics). The contribution of the different OTUs to the group separation was determined by variable importance in projection (VIP) measurements. A VIP score>1 was considered to be an adequate threshold to determine discriminant variables in the PLS-DA model (Wold et al., 2001; Li et al., 2012; Kieffer et al., 2016).

### Metagenome Prediction and Pathway Analysis

In order to infer which pathways were significantly changing among the age groups, Piphillin was used to normalize the amplicon data by 16S rDNA gene copy number and to infer metagenomic contents (Iwai et al., 2016). This analysis was performed using the information of the 89 OTUs (VIPs component 1 + 2 > 1) which showed to significantly contribute to the group separation in the PLS-DA analysis. The raw count-table and the associated 16S rDNA representative sequences were submitted to Piphillin. For the analysis, a sequence identity cut-off of 97% was implemented, and the inferred metagenomic functions were assigned using the Kyoto Encyclopedia of Genes and Genomes database (KEGG; Oct2018 Release). Raw KEGG pathway output from Piphillin was analyzed by DESeq2 using default parameters, after flooring fractional counts to the nearest integer (Love et al., 2014; Bledsoe et al., 2016). The inferred metagenomic pathways were considered differentially represented using a FDR-corrected significance threshold of 0.05.

## Results

### Biometric Data

For this study, fish of 1-, 2- and 4-years-old were used. As expected, standard length and body, liver, and intestine weights differed significantly among age groups (Table 1). Necropsy confirmed that all 1-year-old individuals were males, whereas the ones belonging to the 2- and 4-year-old groups were females. Condition factor and hepatosomatic index were significantly lower in 4-year-old animals, whereas the intestine weight index and intestine length index were significantly higher in 1-year-old fish, when compared to the other two groups.

**TABLE 1:** Biometric data (mean ± SEM) of 10 1-, 2-, and 4-year-old fish (Y + 1, Y + 2, and Y + 4, respectively).

	**Age-class**			**ANOVA *P*-value**
	**Y + 1**	**Y + 2**	**Y + 4**	
		
**Sex**	**Male**	**Female**	**Female**	
Body weight (g)	407.4 ± 11.9^a^	870.6 ± 31.4^b^	2487.4 ± 74.3^c^	<0.0001
Standard length (cm)	24.30 ± 0.24^a^	31.43 ± 0.36^b^	46.30 ± 0.55^c^	<0.0001
Condition factor (CF)^1^	2.83 ± 0.05^a^	2.79 ± 0.05^a^	2.51 ± 0.05^b^	<0.0001
Liver weight (g)	4.06 ± 0.25^a^	7.77 ± 0.58^b^	17.39 ± 0.8^c^	<0.0001
Hepatosomatic index (HSI)^2^	0.99 ± 0.05^a^	0.9 ± 0.06^a^	0.7 ± 0.02^b^	0.001
Intestine weight (g)	7.53 ± 0.18^a^	11.25 ± 0.4^b^	27.07 ± 1.16^c^	<0.0001
Intestine weight index (IWI)^3^	1.86 ± 0.07^a^	1.3 ± 0.05^b^	1.09 ± 0.04^c^	<0.0001
Intestine length (cm)	25.38 ± 1.14^a^	26.54 ± 1.77^a^	35.20 ± 1.64^b^	0.0002
Intestine length index (ILI)^4^	104.7 ± 5.1^a^	84.29 ± 5.3^b^	76.04 ± 3.5^b^	0.0007

*Row values with different superscript letters (a, b, and c) indicate significant differences among age groups in each pairwise comparison (ANOVA + Holm–Sidak test, *P*-value < 0.05). ^1^CF = 100 × (body weight/standard length^3^). ^2^HSI = 100 × (liver weight/fish weight). ^3^IWI = 100 × (intestine weight/fish weight). ^4^ILI = 100 × (intestine length/fish length).*

### Microbiota Diversity and Composition

A total of 686,461 high-quality reads, corresponding to an average of 22,882 reads per sample (for more details, see Supplementary Table 1) were assigned to 846 OTUs at 97% identity threshold. Rarefaction curves approximated saturation (horizontal asymptote), thus a good coverage of the bacterial community was achieved and the number of sequences for analysis was considered appropriate (Supplementary Figure 1). Out of the 846 OTUs, 28.96% were classified up to the level of species, 72.93% to the level of genus, 89.36% to the level of family and more than 90% to the levels of order (93.74%), class (97.28%), and phylum (98.82%). Krona analyses presented in Supplementary Figures 2–4 allow for a complete visual exploration of the relative abundances of the different OTUs in the different age groups.

**FIGURE 1:**
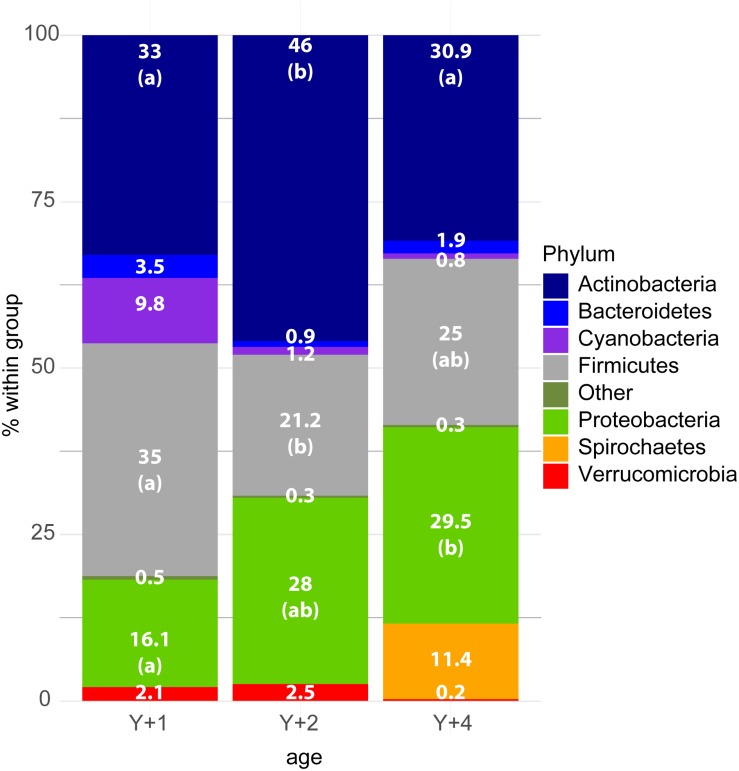
Stacked bar chart representing the relative abundance of bacterial phyla in the different age groups. *Y* + 1, *Y* + 2, and *Y* + 4 correspond to 1-, 2- and 4-year-old gilthead sea bream, respectively. The Kruskal–Wallis test (post-test Dunn’s, *P*-value < 0.05) showed significant differences among groups for the phyla Actinobacteria, Firmicutes, and Proteobacteria. The differences are indicated by different letters in parenthesis which correspond to pairwise comparisons within each phylum among age groups.

The diversity and richness of the bacterial populations did not differ among the different age groups (Table 2). However, differences were found in bacterial composition. Already at the level of phylum, significant differences were found among groups (Figure 1). Actinobacteria, Firmicutes, and Proteobacteria were the most abundant phyla in all age-classes, and the relative abundance of these three phyla was different among groups. In 2-year-old animals, Actinobacteria were significantly more abundant when compared to the other two groups, whereas Firmicutes appeared in significantly lower proportion. Proteobacteria increased in abundance with age, being significantly more abundant in 4-year-old animals when compared to 1-year-olds, with 2-year-olds appearing in the middle. Spirochetes appeared in a significant proportion (11.4%) only in 4-year-old animals, being absent from the other groups.

**TABLE 2:** Species richness estimates (Chao1 and ACE) and diversity indexes (Shannon and Simpson) of 10 fish of 1-, 2-, and 4-year-old (Y + 1, Y + 2, and Y + 4, respectively).

	**Age-class**			**K–W test *P*-value**
	**Y + 1**	**Y + 2**	**Y + 4**	
Chao1	100.7 ± 15.46	121.8 ± 7.69	116.4 ± 21.29	0.242
ACE	102.2 ± 13.57	129.7 ± 8.65	119.9 ± 18.85	0.241
Shannon	2.23 ± 0.16	2.34 ± 0.11	2.24 ± 0.29	0.875
Simpson	0.79 ± 0.05	0.81 ± 0.02	0.76 ± 0.08	0.910

*No statistically significant differences were found among different age groups (K–W test, Kruskal–Wallis test).*

### Core Microbiota

**FIGURE 2:**
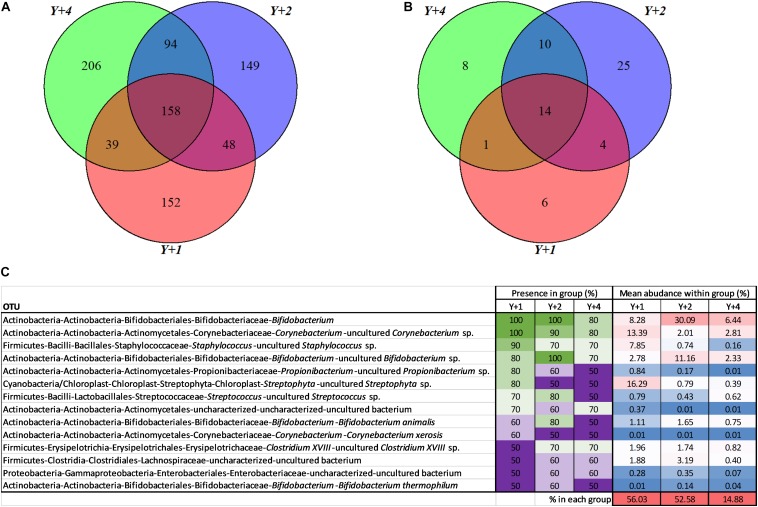
Core microbiota. **(A)** Venn diagram depicting unique and shared OTUs among different age groups. **(B)** Venn diagram depicting unique and shared OTUs among different age groups including only OTUs that are present at least in 50% of the samples of each group. **(C)** Abundance table representing the core 14 OTUs from **(B)**. The presence within each group, in percentage, is highlighted with a color scale with green being the highest and purple the lowest. The mean abundance of each OTU within each group, in percentage, is highlighted with a color scale being red the highest and blue the lowest. The last row represents the percentage of the total microbiota per group constituted by the core microbiota. *Y* + 1, *Y* + 2, and *Y* + 4 correspond to 1-, 2- and 4-year-old gilthead sea bream, respectively.

From the 846 OTUs, 158 were found in at least one individual of the three groups (Figure 2A). The groups that had more OTUs in common were 4- and 2-year-olds (252), whereas 1-year-olds shared 206 OTUs with 2-year-olds, and 197 with 4-year-olds. The use of pools was avoided due to the high individual variability within and among groups. To have a representative overview, a subsequent analysis was limited to OTUs that were present in at least 50% of the individuals of each group (Figure 2B). In this analysis, 2- and 4- year-old animals again shared the largest number of OTUs (24), followed by 2- and 1- year-olds (18). Four- and 1-year-old animals shared the lowest number of OTUs (15). Overall, 14 OTUs constituted what we considered the true core microbiota (shared by more than 50% of individuals from each group). This true core microbiota constituted more than 50% of the total microbial composition in 1- and 2-year-old groups, but decreased to a 15% in 4-year-olds (Figure 2C). The most abundant genera within this true core were *Bifidobacterium*, *Corynebacterium*, and *Staphylococcus*.

### Detailed Microbial Composition

**FIGURE 3:**
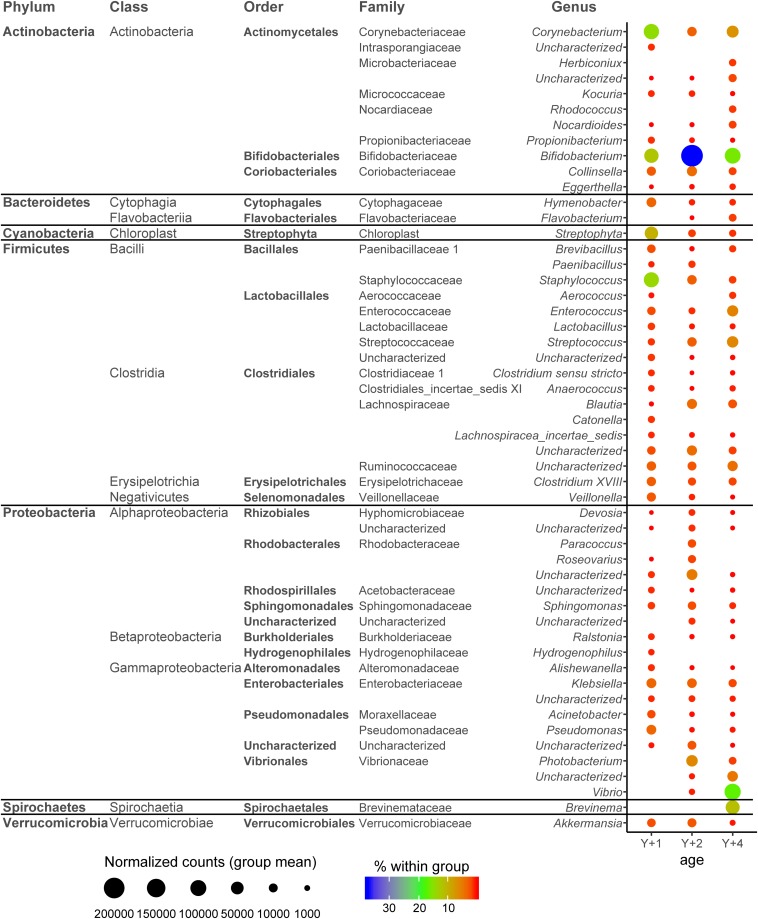
Dotplot map depicting the genera with more than 2,500 normalized counts in at least one age group. The size of the dots represents the normalized counts in each group. The color scale represents the mean abundance, in percentage, of each genus within each group. *Y* + 1, *Y* + 2, and *Y* + 4 correspond to 1-, 2- and 4-year-old gilthead sea bream, respectively.

To have an overview of the microbial composition of the different age groups, we studied the genera that were present with more than 2,500 normalized counts (0.5% of total counts in the sample) in at least one age group (Figure 3), which constituted 94.2, 94.5, and 94% of the total bacterial composition in 1-, 2-, and 4-year-old groups, respectively. For a OTU to be considered it had to be present in at least four individuals per group. The most abundant Actinobacteria genera were *Corynebacterium* (highest in 1-year-olds, 14.7% of all bacteria within this group) and *Bifidobacterium* (highest in 2-year-olds, 37.5%). *Hymenobacter* was the only Bacteroidetes genus present in all age groups, and its abundance decreased with age. The Cyanobacteria genus *Streptophyta* was considerably abundant in 1-year-old fish (9.8%). The phylum Firmicutes was represented by a large number of genera in all age groups, mainly from the classes Bacilli (orders Bacillales and Lactobacillales) and Clostridia (order Clostridiales). The highest represented Firmicutes genus was *Staphylococcus*, constituting 14% of the total bacteria in 1-year-olds, but significantly decreasing in abundance with age (2.9% in 2-year-olds and 1.1% in 4-year-olds). Other abundant Firmicutes genera were *Enterococcus* and *Streptococcus*, reaching more than 5% in abundance in 4-year-olds, slightly decreasing in the other two groups of age. The class Alphaproteobacteria represented 12.2% of the microbiota of 2-year-old fish, whereas it was much less abundant in 1- and 4-year-old animals (2.3 and 0.73%, respectively). The families Rhodobacteraceae and Sphingomonadaceae accounted for most of these differences. The overall abundance of Betaproteobacteria in all age groups was low, not reaching abundance percentages higher than 1% in any group. Within the class Gammaproteobacteria, the family Vibrionaceae was highly abundant in 4-year-olds (22.9%) with a 17.1% of bacteria belonging to the *Vibrio* genus. *Photobacterium* was significantly abundant in 2-year-olds (6%), whereas no member of the Vibrionaceae family was present in 1-year-olds. *Klebsiella* (Enterobacteriaceae) was also significantly abundant in 1- and 2-year-olds (>3%), and the Pseudomonadales *Pseudomonas* and *Acinetobacter* in 1-year-olds (3.4 and 1.8%, respectively). The genus *Brevinema* (Spirochetes) constituted 11.4% of the microbial population of 4-year-old animals, being absent from the other age groups.

### Beta Diversity and Discriminant Analysis

**FIGURE 4:**
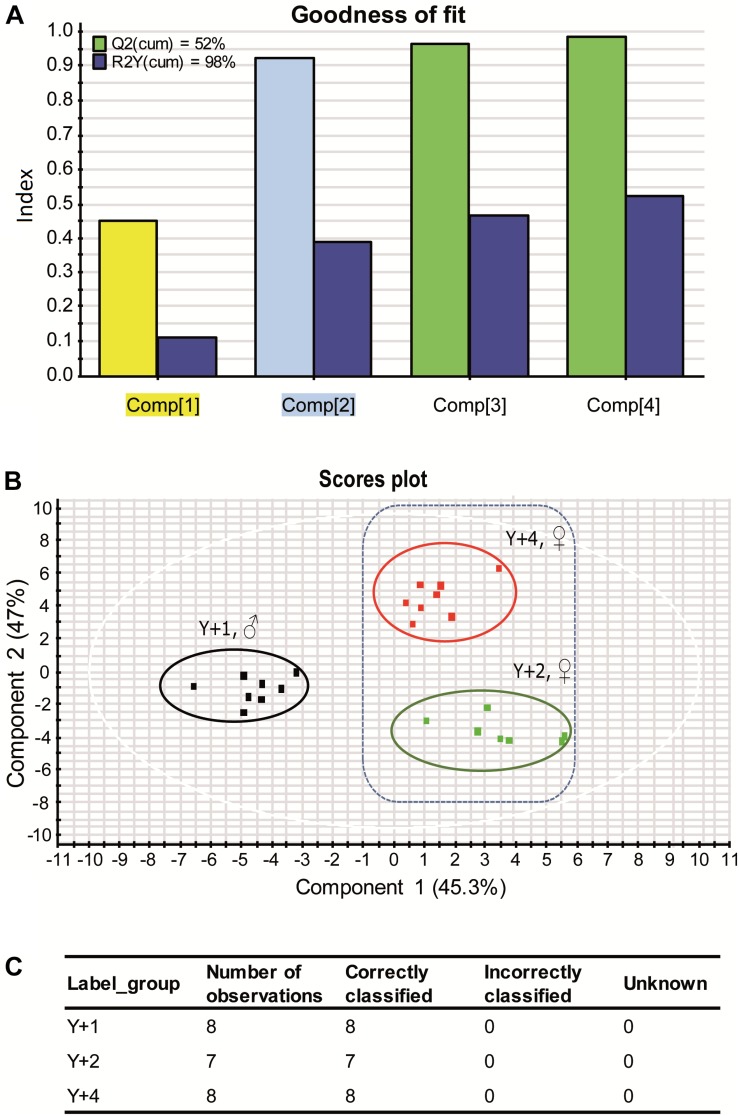
**(A)** Graphical representation of the goodness-of-fit of the PLS-DA model. Colors of highlighted components in *X* axis correspond to the colors of the VIP values in Figure 5B. **(B)** Two-dimensional PLS-DA score plot representing the distribution of the samples between the first two components in the model. **(C)** Classification summary per group provided by EZinfo v3.0 software showing the suitability of the samples.

**FIGURE 5:**
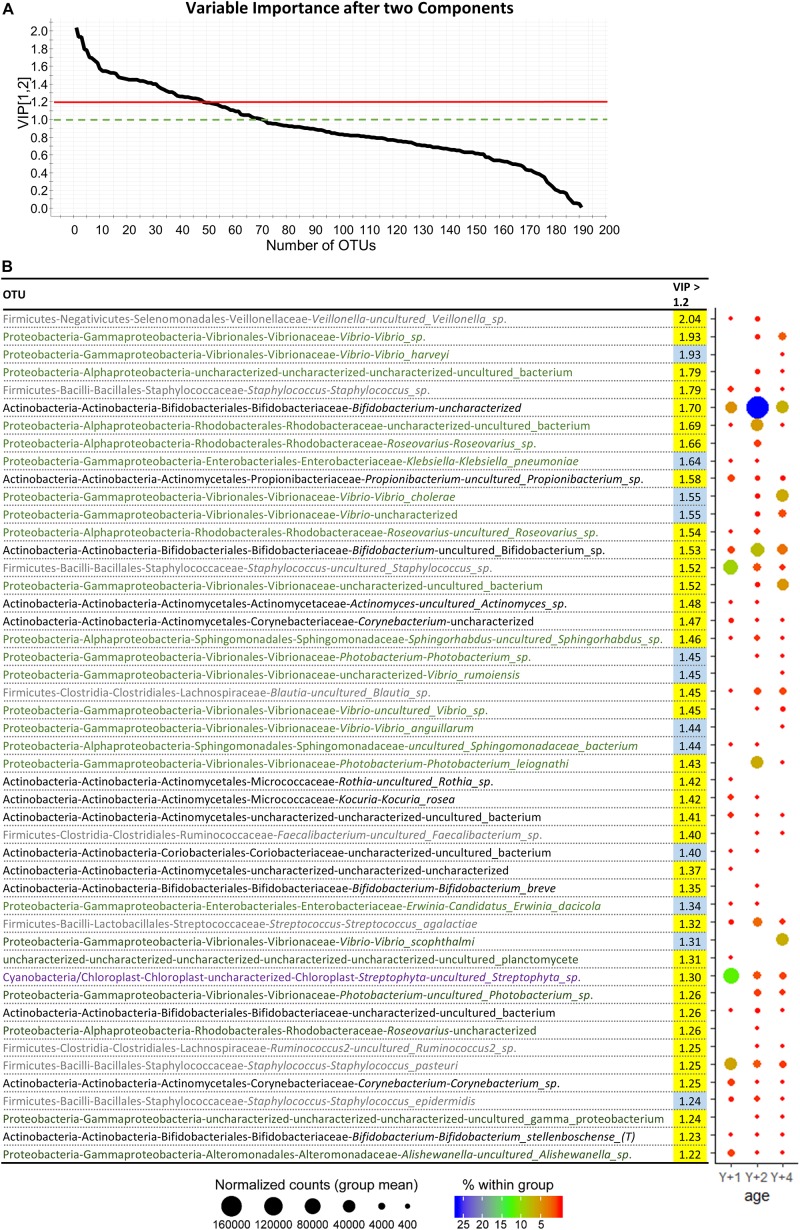
**(A)** Graphical representation of the variable importance (VIP) scores after component 2. **(B)** Dotplot map of OTUs with variable importance in projection (VIP) > 1.2 after two components. VIP values in yellow represent the ones with importance in component 1, whereas the ones in blue appear after component 2. The size of the dots represents the normalized counts in each group. The color scale represents the abundance, in percentage, of each genus within each group. *Y* + 1, *Y* + 2, and *Y* + 4 correspond to 1, 2- and 4-year-old gilthead sea bream, respectively.

Despite the high variability within groups, beta diversity among age groups was significantly different (PERMANOVA, pseudo *F*-ratio = 0.0039). The discriminant model (PLS-DA) was based on four components, which explained 98% [R2Y (cum)] and predicted 52% [Q2 (cum)] of the total variance (Figure 4A). During the statistical processing to construct the model, two fish from the 1-year-old group, three from the 2-year-old group, and two from the 4-year-old group appeared as outliers and were excluded from the model. The fit of the PLS-DA model was validated using a permutation test (Supplementary Figure 5). A clear separation by sex was observed along the first component, whereas component 2 separated groups by age (Figure 4B). According to the obtained misclassification table (Figure 4C), all samples were properly classified in their respective groups. In component 1, the number of OTUs with significant VIP values (>1) was 72, 50 of which had a VIP > 1.2 and are shown in Supplementary Figure 6 with their corresponding abundances per age group. When the second component was also considered, 71 OTUs had VIP > 1 (Supplementary Table 2), with 48 OTUs with VIP > 1.2 (Figure 5). The OTUs driving the separation along components 1 and 2 mainly belonged to the phyla Firmicutes, Proteobacteria and Actinobacteria. More specifically, *Veillonella*, *Staphylococcus* (Firmicutes), *Propionibacterium*, *Actinomyces*, and *Corynebacterium* (Actinobacteria) were decreasing gradually with age, whereas *Bifidobacterium* (Actinobacteria) and the family Rhodobacteraceae (Proteobacteria) had a higher dominance in 2-year-olds. The Proteobacteria genus *Klebsiella* decreased gradually with age, whereas several OTUs of the *Vibrio* genus appeared in 2-year-olds and increased with age.

### Pathway Analysis

In an attempt to evaluate the biological significance of the microbiota changes among age groups, KEGG pathway analysis was performed using the inferred metagenome constituted by the 89 OTUs with significant VIP values in components 1 and 2. Of note, this analysis only provides information about genes that could potentially be expressed in these populations, which would inform about the possible metabolic capacity of the intestinal bacteria present in each group. Further studies on proteomics and metatranscriptomics will reveal the actual pathways present in these populations.

Differentially abundant (FDR corrected) inferred metagenomics functions were mainly found between 4- and 1-year-old metagenomes (Table 3). Only one pathway (flagellar assembly) was differentially abundant between 2- and 1-year-old groups, whereas no differences were found between 2- and 4-year-old animals. Pathways related to cell death (apoptosis and ferroptosis), cell motility (flagellar assembly and chemotaxis), biofilm formation, environmental response (two-component system) and infection were significantly up-regulated in 4-year-old’s microbiota. Pathways related to metabolism were the largest represented among the differentially expressed pathways. Secondary metabolites, glycosphingolipids, lipids and ansamycins biosynthesis, cyanoaminoacid metabolism, and nitrotoluene degradation were up-regulated in 4-year-old animals, whereas carbohydrate and lipoic acid metabolism and atrazine degradation were significantly down-regulated.

**TABLE 3:** Pathway analysis from predicted metagenome.

**Class**	**Pathway**	**log2fc**	**SEfc**	***P*adj**
Cellular processes; Cell growth and death	Apoptosis	5.59	1.79	0.021
	Ferroptosis	0.71	0.21	0.010
Cellular processes; Cell motility	Flagellar assembly^∗^	8.92	1.26	< 0.001
	Bacterial chemotaxis	4.04	0.68	< 0.001
Cellular processes; Cellular community – prokaryotes	Biofilm formation – *Pseudomonas aeruginosa*	1.35	0.42	0.014
	Biofilm formation – *Vibrio cholerae*	1.33	0.33	0.001
Environmental information processing; Signal transduction	Two-component system	0.57	0.15	0.004
Human diseases; Infectious diseases: Bacterial	*Vibrio cholerae* infection	10.86	1.66	< 0.001
	*Salmonella* infection	1.65	0.57	0.033
Metabolism; Biosynthesis of other secondary metabolites	Biosynthesis of secondary metabolites – unclassified	1.02	0.31	0.011
Metabolism; Carbohydrate metabolism	Propanoate metabolism	–0.20	0.07	0.042
	Glyoxylate and dicarboxylate metabolism	–0.26	0.09	0.031
	Glycolysis/Gluconeogenesis	–0.36	0.13	0.045
	Citrate cycle (TCA cycle)	–0.41	0.13	0.020
Metabolism; Glycan biosynthesis and metabolism	Glycosphingolipid biosynthesis – globo and isoglobo series	1.69	0.38	< 0.001
Metabolism; Lipid metabolism	Steroid hormone biosynthesis	3.12	1.11	0.042
	Sphingolipid metabolism	2.06	0.50	0.001
Metabolism; Metabolism of cofactors and vitamins	Lipoic acid metabolism	–1.12	0.32	0.009
Metabolism; Metabolism of other amino acids	Cyanoamino acid metabolism	0.50	0.17	0.026
Metabolism; Metabolism of terpenoids and polyketides	Biosynthesis of ansamycins	1.31	0.39	0.010
Metabolism; Xenobiotics biodegradation and metabolism	Nitrotoluene degradation	3.57	0.71	< 0.001
	Atrazine degradation	–1.71	0.57	0.027

*Piphillin inferred KEGG pathways significantly different (*P*adj < 0.05) between age groups *Y* + 4 and *Y* + 1. log2fc indicates the log2 fold change in the comparison *Y* + 4 vs. *Y* + 1. SEfc is the standard error of the calculated fold change.^∗^The flagellar assembly pathway was also found significantly different between age group *Y* + 2 and *Y* + 1 (*Y* + 2 vs. *Y* + 1: lof2fc = 6.65, SEfc = 1.56, *P*adj = 0.006). No significant differences were found between *Y* + 2 and *Y* + 4.*

## Discussion

In all animals, intestinal microbial colonization starts at birth or hatching. Early colonization is related to environmental microbiota and feeding and has key consequences in intestinal function and immune development. Several studies have addressed the changes in intestinal microbiota of fish during ontogenic development (Romero and Navarrete, 2006; Ingerslev et al., 2014; Bakke et al., 2015; Bledsoe et al., 2016; Stephens et al., 2016). These studies conclude that, as a general rule, the intestinal microbiota at early developmental stages exhibits higher interindividual variability and greater sensitivity to alteration. As in humans (Yatsunenko et al., 2012), the diversity of bacteria increases as fish develop (Egerton et al., 2018), whereas, older animals host relatively stable populations due to the homeostatic equilibrium acquired in adults. However, in humans, age- and sex-dependent differences in intestinal microbiota were found in adults, with women having higher diversity than men (de la Cuesta-Zuluaga et al., 2019). In fish, several studies demonstrate sex-specific differences in adult intestinal microbiota which are attributed to the different sex hormones, but the exact mechanisms remain to be studied (Chen et al., 2018a, b; Zha et al., 2018). These studies emphasize the importance of including age and sex information when analyzing the microbiota of any species. The current work is, to our knowledge, the first study aimed to determine changes in intestinal bacterial populations at different stages of gilthead sea bream adulthood. The results obtained manifested that while the bacterial diversity, in terms of richness estimates and diversity indexes, remains constant through the different age and sex groups, there are significant and gradual changes in bacterial composition out of the reproductive season of gilthead sea bream, that spans in our experimental facilities between December and January (Simó-Mirabet et al., 2018).

The phyla Proteobacteria, Bacteroidetes, and Firmicutes represent up to 90% of fish intestinal microbiota in different species (Ghanbari et al., 2015). In gilthead sea bream in particular, the dominant phyla described depend on the study. A high prevalence of Firmicutes, Proteobacteria, and Bacteroidetes was found in non-adherent bacteria of gilthead sea bream stomach and intestine (Silva et al., 2011), whereas Proteobacteria was found to constitute around 80% of the adherent microbiota in posterior intestine (Piazzon et al., 2017). Other studies showed that Actinobacteria, Firmicutes, Proteobacteria, and Bacteroidetes dominated the autochthonous intestinal microbiota of gilthead sea bream (Kormas et al., 2014) and the same phyla were found predominant in the anterior intestinal segment (Estruch et al., 2015), which coincides with the experimental design and results of the current study. Parallel to our results, Kormas et al. (2014) reported a greater presence of Alpha- and Gammaproteobacteria over Betaproteobacteria. Cyanobacteria sequences are often removed from these types of analyses as they are considered contaminants from the environment and food. However, in the current study we decided to keep these sequences because, despite intestines were thoroughly washed to remove non-adherent material, the abundance of Cyanobacteria in 1-year-old animals was significant, constituting almost 10% of the total bacteria. Recently, a whole genome reconstruction of Cyanobacteria found in human fecal samples led to the proposal of a new candidate phylum sibling to Cyanobacteria named Melainabacteria. Melainabacteria are non-photosynthetic obligate anaerobic fermenters that can ferment plant polysaccharides in the gut providing the host with vitamins B and K and are mutualistic components of the intestinal bacterial communities in mammals (Di Rienzi et al., 2013). A previous study on gilthead sea bream which retained Cyanobacteria sequences during the analysis (Parma et al., 2017), also found Cyanobacteria as part of the bacterial communities of the intestinal microbiota. Whether these bacteria are contaminants or true symbionts remains to be studied. However, their high prevalence within the adherent microorganisms seems to indicate a role for these bacteria in the intestinal physiology of gilthead sea bream.

Actinobacteria are widely distributed in terrestrial and aquatic environments and are known to form symbiotic interactions with vertebrates and invertebrates taking part in host health by converting the feedstuffs into microbial biomass and fermentation end products that can be utilized by the host (Anandan et al., 2016). Coinciding with the results of our comparisons between 1- and 2-year-old group, the proportion of Actinobacteria in the intestines of adult control female zebrafish is higher than in males (Chen et al., 2018a). The main Actinobacteria species characterized in gilthead sea bream intestine belonged to the genera *Corynebacterium*, *Propionibacterium*, and *Bifidobacterium* (Kormas et al., 2014; Estruch et al., 2015; Nikouli et al., 2018; Castro et al., 2019) which are part of the core microbiota described in the current study. These three genera, together with *Kocuria*, *Actinomyces*, and *Rothia* were also part of the species driving the separation by age and sex in our discriminant analysis. Of note, all the previous studies in gilthead sea bream used animals between 260 and 451 g, which would be equivalent to our 1-year-old male group. None of these previous studies reported the sex of the animals studied. *Corynebacterium* species are known to contribute to manganese acquisition and produce superoxide dismutase and lipases to form organic fatty acids and thioalcohols (Grice, 2014; Johnson et al., 2018). This genus showed a higher presence in rainbow trout intestinal microbiota when fish were fed high lipid diets (Huyben et al., 2019). The production of superoxide dismutase, besides being a mechanism of self-protection, can also prevent oxidative damage to host tissues; and the scavenging of manganese may inhibit colonization by other microbes (Cogen et al., 2008). *Kocuria* species are normally found in fish gastrointestinal tracts and have been proposed to be used as a probiotic in aquaculture due to their capacity to grow in a wide range of temperature, salinity and pH, and their production of extracellular enzymes that may have a role in digestion (Sharifuzzaman et al., 2018). Several reports state that *Propionibacterium* are rarely associated with fish (Dehler et al., 2017; Egerton et al., 2018). However, they seem to be an important part of the core microbiota of gilthead sea bream (Kormas et al., 2014; Estruch et al., 2015; Nikouli et al., 2018). The genus *Propionibacterium* has a unique metabolism being able to synthesize propionate, linolenic acid and vitamins, remove toxic and antinutritional compounds from the environment, and produce antimicrobials (Zárate, 2012). Propionate is a short chain fatty acid that, together with butyrate – another microbial byproduct from the digestion of carbohydrates – has shown to be very beneficial for intestinal health. These microbial metabolites are an important source of energy, induce strengthening of the epithelial barrier, reduce inflammation and increase the production of mucus and antimicrobial peptides (Onrust et al., 2015; Piazzon et al., 2017). Indeed, short chain fatty acids have extensively shown their benefits on gilthead sea bream intestinal health (Estensoro et al., 2016; Piazzon et al., 2017). The current results show a decreasing trend with age of these three genera – *Corynebacterium*, *Kocuria*, and *Propionibacterium* – which can indicate that bacterial communities in mature female fish have less capacity to produce organic fatty acids, vitamins and microbial inhibitors. An opposite trend was found for *Bifidobacterium*, which were very abundant in all age groups but are particularly abundant in 2-year-old animals. Bifidobacteria are not commonly found in fish, however, some studies have described their presence in some fish species (Vlková et al., 2012), including gilthead sea bream (Castro et al., 2019). In our analysis we detected more than 10 species of *Bifidobacterium*, of which *Bifidobacterium thermophilum* and *Bifidobacterium animalis* are part of the core microbiota for all age groups, and *Bifidobacterium breve* and *Bifidobacterium stellenboschense* are part of the significant VIPs driving the separation by age and sex. Bifidobacteria are known for their potential as a probiotic in humans due to their health promoting properties such as fatty acid production, inhibition of pathogens, and immunostimulatory role (Russell et al., 2011). The current results also support their beneficial activities in gilthead sea bream and their increase with age could be a means to metabolically compensate the decrease of other Actinobacteria.

Firmicutes are very common bacteria in the intestine of fish and mammals (Lozupone et al., 2012; Ghanbari et al., 2015). Their prevalence in gilthead sea bream intestine is often quite high (Estruch et al., 2015; Parma et al., 2017) and their abundance is highly modulated by dietary interventions (Piazzon et al., 2017). The genera previously described as abundant in gilthead sea bream intestine were *Lactobacillus*, *Clostridium*, *Streptococcus*, *Staphylococcus*, and *Veillonella* (Estruch et al., 2015; Parma et al., 2017; Nikouli et al., 2018). Our current results show that *Clostridium*, *Staphylococcus*, and *Streptococcus* are particularly abundant and are part of the core microbiota of gilthead sea bream adherent microbial communities of the anterior intestine. In addition, the genera *Blautia*, *Veillonella*, and *Faecalibacterium* are also part of the bacteria significantly changing among age and sex groups. Clostridia species are extremely heterogeneous and can be symbiont or pathogenic. They are classified in 19 clusters, and cluster XVIII – part of the core microbiota found in the current study– regulates intestinal T cell populations (Narushima et al., 2014), which confers them a critical role in host immunity. They also contribute to the host’s nutrition by producing short chain fatty acids (butyrate) and vitamins. *Staphylococcus* species can be pathogenic or mutualistic, with important roles in pathogen inhibition and immune training (Cogen et al., 2008). *Blautia* and *Faecalibacterium* are short chain fatty acid producing bacteria (acetate and butyrate, respectively) (Meng et al., 2018). Both genera increase with age, in particular *Faecalibacterium* which is not present in 1-year-old male animals. Their increase with age could be linked to a metabolic compensation for the decrease of other short chain fatty acid producing bacteria, as it was hypothesized before for *Bifidobacterium*.

Proteobacteria significantly increased with age and showed the highest variability among age classes, to the extent that, even though they were always present in high percentages, no specific Proteobacteria was characterized in the core microbiota at the genus or species level. This heterogeneity of the Proteobacteria population in gilthead sea bream gastrointestinal tract was also found in previous studies (Estruch et al., 2015). Nonetheless, it is undeniable that Proteobacteria, as a phylum, are an important part of the core microbiota of this species. Our study showed that Proteobacteria significantly and gradually increased with age and were consequently higher in females, with Alpha- and Gammaproteobacteria being higher in 2- and 4-year-old female animals, respectively. Interestingly, zebrafish adult females showed higher abundance of intestinal Proteobacteria than males (Chen et al., 2018b). In our study, the genera *Alishewanella*, *Klebsiella*, and *Erwinia* gradually decreased with age, whereas the family Vibrionaceae, particularly the genera *Photobacterium* and *Vibrio*, dramatically increased in older animals. The complete absence of Vibrionaceae in 1-year-old male animals was found of particular interest. Previous studies showed that the inclusion of vegetable ingredients in the diet of this carnivorous fish induced an increase in *Photobacterium* and an increase in disease susceptibility (Estruch et al., 2015; Piazzon et al., 2017). *Photobacterium* and *Vibrio* are common symbionts of fish microbiota that can produce chitinase, amylase, lipase and proteases helping with digestion, but many of these species can produce harmful enzymes and act as pathogens (Egerton et al., 2018). *Vibrio harveyi*, *Vibrio anguillarum*, *Vibrio scophthalmi*, and *Vibrio rumoiensis* appeared in 4-year-old animals, being all, except the latest, causative agents of fish pathologies (Pujalte et al., 2003; García-Aljaro et al., 2012; Castillo et al., 2017). In the current study, all fish were healthy, but the presence of a higher load of opportunistic pathogens can lead to a higher risk of infection under adverse circumstances.

Due to the particular biology of this species, it is difficult to determine whether the changes detected were owed to the age, size or sex of the fish (all individuals from the same age group were of the same sex). However, it is undisputable that adult gilthead sea bream undergo shifts in their intestinal microbiota that are correlated with age/sex and independent of other factors such as diet, environment, season, etc. The microbiota of healthy adults is expected to be relatively stable and the current results show that, even though many changes were detected at OTU level, at a functional level, changes could be fewer due to metabolic compensation. The particular role of the different bacterial populations on their fish host health is poorly studied. The current work aimed to go a step further from just the description of the population, so a more mechanistic view was attempted by inferring the potential pathways that could be over- or underrepresented in the different age groups.

The results of the inferred metagenomes showed a gradual change in the potential pathways for gilthead sea bream intestinal microbiota with age. Significant changes were almost exclusively found when comparing 1- with 4-year-old animals, whereas the 2-year-old group almost did not show differences with any of the other age groups. This prediction showed that older gilthead sea bream could have significantly higher representation of pathways related with pathogenesis and inflammation (i.e., cell death, motility, biofilm formation, and infection). Increased bacterial motility (chemotaxis and flagellar assembly) has been linked to intestinal inflammation in mammalian models (Zheng et al., 2017); and biofilm formation protects against antimicrobial compounds, allowing the growth of pathogens (Dubois et al., 2019). Many bacterial components act as an alarm system in the host, inducing cell death mechanisms to avoid replication and dissemination of pathogens (Giogha et al., 2014).

The key bacterial fermentation products from dietary carbohydrates are short chain fatty acids (Rowland et al., 2018) with many beneficial effects on the host, as described above. Interestingly, the carbohydrate metabolism pathway was predicted to be underrepresented in older animals in the current study. The glycosphingolipid biosynthesis pathway has been associated in healthy centenarian humans with anti-inflammatory and healthy functions of gut microbiota (Kim et al., 2019). In a fish model, symbiont bacteria derived sphingolipids demonstrated a crucial role in mucosal homeostasis and immune cell populations (Sepahi et al., 2016). The higher presence of these pathways in our older animals indicate that these bacterial populations could have the ability to compensate the predicted inflammatory signals and the downregulated carbohydrate pathways. Other pathways predicted to be changing in older animals were lipoic acid metabolism, production of antimicrobial compounds and capacity to degrade toxic substances. These changes could have an effect on the antioxidant capacities of the intestines, the ability to regulate other bacterial populations and the capability of the host to cope with the environment (Spalding and Prigge, 2010; Claus et al., 2016; Ramírez and Romero, 2017; Tarnecki et al., 2019).

To conclude, the microbial composition of adult gilthead sea bream showed significant changes with age and sex. These changes were also observed when the predicted metabolic capacities of the bacterial populations were compared, finding that older animals could potentially be more prone to an inflammatory profile with higher representation of bacterial infection pathways and lower production of short chain fatty acids. This research sets the bases for the next step, which will be the study of the composition and actual bacterial pathways being expressed in genetically selected fish. The use of metatranscriptomic and proteomic analyses and dietary interactions will help to validate and complete the description of the functionality of the gilthead sea bream intestinal microbiota.

## Data Availability Statement

The datasets generated for this study can be found in the Sequence Read Archive (SRA; https://www.ncbi.nlm.nih.gov/sra) under the Bioproject accession number PRJNA554554 (BioSample accession numbers: SAMN12273608-37).

## Ethics Statement

The animal study was reviewed and approved by the Ethics and Animal Welfare Committee of IATS and CSIC according to national (Royal Decree RD53/2013), and the EU legislation (2010/63/EU) on the handling of animals for experiments.

## Author Contributions

JP-S and AS-B performed conceptualization and funding acquisition. MP, FN-C, PS-M, AP-S, and JC-G participated in the material preparation, and sample collection and processing. FN-C, FR, JP-S, and MP performed data curation and formal analysis. MP, FN-C, and JP-S visualized and wrote the original draft. All authors contributed to the revision and editing of the manuscript.

## Conflict of Interest

FR was employed by the company Biotechvana SL and is currently employed by Imegen. The remaining authors declare that the research was conducted in the absence of any commercial or financial relationships that could be construed as a potential conflict of interest.

## Footnotes

1 http://www.bioinformatics.babraham.ac.uk/projects/fastqc/
