# Supplementary material for: Sex, Age, and Bacteria: How the Intestinal Microbiota Is Modulated in a Protandrous Hermaphrodite Fish
Source: Front Microbiol. 2019 Oct 31;10:2512. doi: 10.3389/fmicb.2019.02512 (PMC6834695; doi:10.3389/fmicb.2019.02512)
Supplement: Supplementary file 2 [file Data_Sheet_2.zip › Figure captions.pdf]

Figure Captions:

**Supplementary Figure 2**. Krona visualization of the relative abundance of intestinal bacterial OTUs identified in 1-year-old fish. (HTML 32 kb)

**Supplementary Figure 3**. Krona visualization of the relative abundance of intestinal bacterial OTUs identified in 2-year-old fish. (HTML 38 kb)

**Supplementary Figure 4**. Krona visualization of the relative abundance of intestinal bacterial OTUs identified in 4-year-old fish. (HTML 38 kb)
