# Supplementary material for: Sex, Age, and Bacteria: How the Intestinal Microbiota Is Modulated in a Protandrous Hermaphrodite Fish
Source: Front Microbiol. 2019 Oct 31;10:2512. doi: 10.3389/fmicb.2019.02512 (PMC6834695; doi:10.3389/fmicb.2019.02512)
Supplement: Supplementary file 2 [file Data_Sheet_2.zip › Supplementary Figure 2_1Y.html]

Javascript must be enabled to view this page.

magnitude
magnitudeUnassigned

T5\_krona

17836

46

46

46

46

17790

552

519

519

519

11

508

508

6

6

6

6

6

27

27

27

27

27

2913

2913

6

1

2906

132

5

5

5

127

127

127

127

127

5662

3777

3061

3

3

3

454

363

363

91

91

2560

3
2560

24

125

1451

23

934

13

31

24
31

7

716
143

3

62

62

31

6

25

13

13

13

4

263

263

25

175

63

177

177

24

12

141

51

51

51

349

349

349

349

349

528

528

528

528

528

1008

1008

2

2

2

23

22

22

1

1

52

52

52

652

5

5

2

102

102

20

336

12

12

5

5

170

170

41

41

41

238

2

14

14

218

4

4

2

7

6070

6070

2263

2263

2

2255
1477

2

281

495

6

3

3

3

3

364

364

364

56

308

4

4

3436

65

1

1

1

1

1

1

5

5

5

159

13

10

3

30

116

21

95

18

149

149

149

10

10

158

20

9

9

129

17

17

17

14

14

14

2839

28
2839

1

103

2546

1

75

52

4

13

10

1

2

3

2414

2096

975

273

273

253

20

702

1

3

11

11

32

655

1

654

898

898

13

13

48

48

5

66

66

4

1

1

672

136

536

5

2

1

1

35

47

58

58

30

28

28

18

18

18

18

29

118

118

118

118

198

154

9

9

15

5

5

10

10

7

121

2

2

119

119

2

2

2

8

8

6

2

36

36

36

36

120

52

52

9

43

43

21

11

11

11

10

10

19

19

19

28

28

3

3

25

4

4

4

1

1

1

3

3

3

34
