# Supplementary material for: Sex, Age, and Bacteria: How the Intestinal Microbiota Is Modulated in a Protandrous Hermaphrodite Fish
Source: Front Microbiol. 2019 Oct 31;10:2512. doi: 10.3389/fmicb.2019.02512 (PMC6834695; doi:10.3389/fmicb.2019.02512)
Supplement: Supplementary file 2 [file Data_Sheet_2.zip › Supplementary Figure 3_2Y.html]

Javascript must be enabled to view this page.

magnitude
magnitudeUnassigned

T26\_krona

16675

16661

16

16

1

1

1

1

1

1

3501

319

319

319

27

27

292

292

899

388

3

3

3

69

6

6

63

63

316

3
316

35

132

120

2

24

511

1

113

113

41

44

9

19

368

358

72

1

285

10

10

22

22

2

20

7

2

2

1

2

2

53

53

53

53

26

27

2230

2230

2

1

1

1

1

696

11

11

576

1

24

84

84

40

34

34

6

2

4

3

2

2

1

1

1481

1

1

57

2

2

19

20

20

788

788

7

7

3

3

11

11

2

2

536

35

35

8

8

8

4

4

4

4

4

4

1

140

14

14

14

14

11

3

6

6

6

4

2

2

116

116

116

73

73

43

43

4

4

2

2

2

2

2

2

145

7
145

4

131

3

12

529

525

525

525

525

525

4

4

4

4

4

3680

1664

1

1

1

10

10

6

6

4

4

185

6

6

6

68

44

44

44

32

32

3

29

35

35

35

1112

1112

5

209
1

208

670

2

2

1

1

223

219

4

2

2

71

1

16

268

256

6

6

3

1

1

11

177

1

176

58

58

6

6

3

3

3

1981

3

216

25

14

14

14

11

3
11

5

3

988

9
988

1

1

887
7

17

30

687

146

1

6
52

12

1

21

12

38

740

740

4

4

55

55

21

21

538

195

340

3

3

58

5

1

3

1

6

14

26

4

4

2

2

2

2

4

4

4

4

1

1

1

4

4

4

4

35

35

9

9

9

2

2

24

14

14

10

8632

8632

2

2

2

2

7320

7320

19

19

22

43

7236
5061

19

278

1877

1

1

1

1

1

441

1

343

343

4

1

338

1

1

1

1

1

1

1

1

4

4

4

4

4

4

2

2

28

28

28

56

49

30

19

5

5

2

2

868

868

830

687

143

16

22

5

17

14

14

14

14
