# Supplementary material for: Sex, Age, and Bacteria: How the Intestinal Microbiota Is Modulated in a Protandrous Hermaphrodite Fish
Source: Front Microbiol. 2019 Oct 31;10:2512. doi: 10.3389/fmicb.2019.02512 (PMC6834695; doi:10.3389/fmicb.2019.02512)
Supplement: Supplementary file 2 [file Data_Sheet_2.zip › Supplementary Figure 4_4Y.html]

Javascript must be enabled to view this page.

magnitude
magnitudeUnassigned

T25\_krona

25520

25

25

25

25

25495

2

2

2

2

31

31

31

31

31

31

2

2

2

2

2

9

10443

10207

9709

9709
57

42

42

171
7024

2

1

733

2

1

3085

2591

435

3

2

167

4

4

11

81

54

10

1

1

1

2417

447

447

32

32

3

3

3

3

1

1

287

87

196

4

2

2

4

4

21

64

60

4

16

3

3

6

3

3

3

40

24

24

24

16

2

2

6

6

8

3

3

3

3

5

5

144

8

8

8

27

27

8

8

2

17

3

14

87

85

4

47

34

13

6

26

26

2

2

2

10

10

7

3

3

12

12

3

3

9

9

3

3

3

3

89

89

2

2

2

82

49

49

2

1

1

26

5

19

2

1

1

3

1

1

1

4

4

4

7336

7336

7336

7336

7336

4069

4069

2547

2547

8

14

1651
2525

597

3

81

193

1189

235

117

2

1

1

6

6

78
62

16

1

24
16

8

3

1

2

2

4

4

4

16

16

16

112

102
13

89

2

2

6

6

2

1

94

3

3

10

10

81

81

2

2

2

725

725
1

1

721

2

2

2

2

2

331

331

5

52

51

1

274

219

55

157

102

102

102

1

1

99
2

94

2

1

2

11

11

2

1

1

1

9

6

6

3

3

35

35

35

35

24

11

9

9

7

7

7

2

2

2

2

2

2

100

100

100

3344

1975

1747

4

2

52

21
52

31

26

26

1

7

18

582

1

581
2

120

314

8

137

1072

1071

1

20

158

892

1

1

9

3

3

3

3

228

7

7

7

124

124

18

39

65

2

90

90

90

7

7

3

16

16

16

3

3

13

13

215

215

215

2

2

2

2

211

211

1135

1135

526

4

2

2

29

29

491

551

8

8

373

373

39

21

1

1

8

8

101

42

42

42

3

3

3

2

2

2

4

7

1

1

6
