# Supplementary figures and images for: Sex, Age, and Bacteria: How the Intestinal Microbiota Is Modulated in a Protandrous Hermaphrodite Fish
Source: Front Microbiol. 2019 Oct 31;10:2512. doi: 10.3389/fmicb.2019.02512 (PMC6834695; doi:10.3389/fmicb.2019.02512)

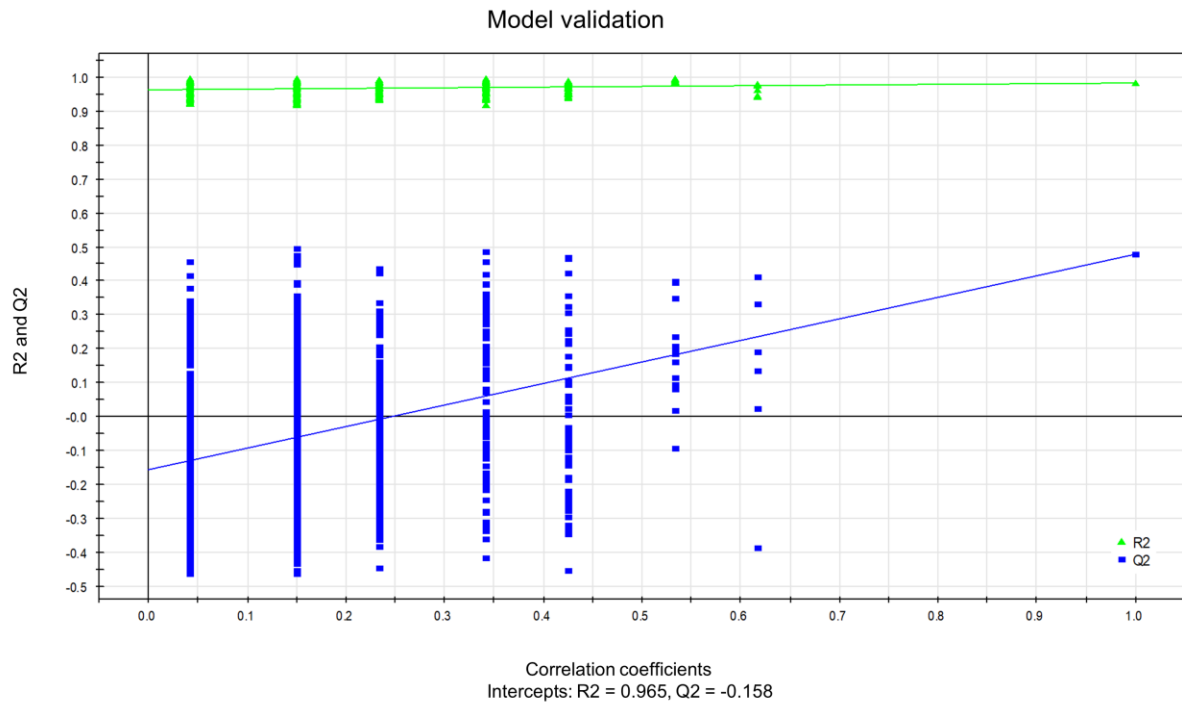

**Supplementary Figure 5** | Validation of the PLS-DA model consisting in 999 random permutations.

Supplement: Supplementary file 3 [file Data_Sheet_3.PDF]
