# Supplementary material for: Sex, Age, and Bacteria: How the Intestinal Microbiota Is Modulated in a Protandrous Hermaphrodite Fish
Source: Front Microbiol. 2019 Oct 31;10:2512. doi: 10.3389/fmicb.2019.02512 (PMC6834695; doi:10.3389/fmicb.2019.02512)
Supplement: Supplementary file 4 [file Data_Sheet_4.PDF]

A

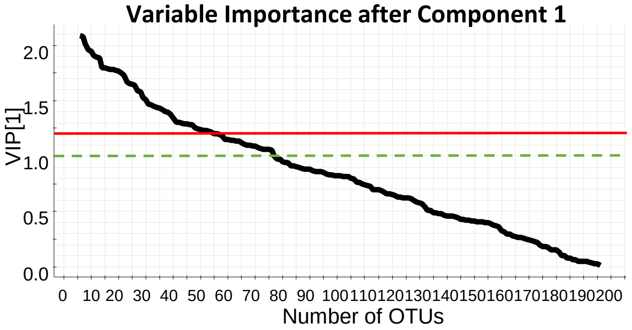

B

OTU	VIP > 1.2
Actinobacteria-Actinobacteria-Actinomycetales-Corynebacteriaceae-*Corynebacterium*-uncharacterized	2.09
Firmicutes-Bacilli-Bacillales-Staphylococcaceae-*Staphylococcus*-*Staphylococcus*_sp.	2.08
Actinobacteria-Actinobacteria-Actinomycetales-uncharacterized-uncharacterized-uncultured_bacterium	2.01
Actinobacteria-Actinobacteria-Actinomycetales-Micrococcaceae-*Rothia*-uncultured_*Rothia*_sp.	1.96
Actinobacteria-Actinobacteria-Bifidobacteriales-Bifidobacteriaceae-*Bifidobacterium*-uncultured_*Bifidobacterium*_sp.	1.94
Actinobacteria-Actinobacteria-Actinomycetales-Micrococcaceae-*Kocuria*-*Kocuria*_*rosea*	1.91
Actinobacteria-Actinobacteria-Actinomycetales-uncharacterized-uncharacterized-uncharacterized	1.89
Proteobacteria-Alphaproteobacteria-uncharacterized-uncharacterized-uncharacterized-uncultured_bacterium	1.89
uncharacterized-uncharacterized-uncharacterized-uncharacterized-uncharacterized-uncultured_planctomycete	1.80
Firmicutes-Negativicutes-Selenomonadales-Veillonellaceae-*Veillonella*-uncultured_*Veillonella*_sp.	1.79
Actinobacteria-Actinobacteria-Bifidobacteriales-Bifidobacteriaceae-*Bifidobacterium*-uncharacterized	1.79
Proteobacteria-Alphaproteobacteria-Rhodobacterales-Rhodobacteraceae-uncharacterized-uncultured_bacterium	1.78
Actinobacteria-Actinobacteria-Actinomycetales-Propionibacteriaceae-*Propionibacterium*-uncultured_*Propionibacterium*_sp.	1.78
Cyanobacteria/Chloroplast-Chloroplast-uncharacterized-Chloroplast-*Streptophyta*-uncultured_*Streptophyta*_sp.	1.77
Actinobacteria-Actinobacteria-Actinomycetales-Corynebacteriaceae-*Corynebacterium*-*Corynebacterium*_sp.	1.77
Firmicutes-Bacilli-Bacillales-Staphylococcaceae-*Staphylococcus*-uncultured_*Staphylococcus*_sp.	1.75
Proteobacteria-Gammaproteobacteria-Vibrionales-Vibrionaceae-*Photobacterium*-uncultured_*Photobacterium*_sp.	1.73
Proteobacteria-Gammaproteobacteria-Vibrionales-Vibrionaceae-*Photobacterium*-*Photobacterium*_*leiognathi*	1.67
Proteobacteria-Gammaproteobacteria-Vibrionales-Vibrionaceae-*Vibrio*-uncultured_*Vibrio*_sp.	1.65
Proteobacteria-Gammaproteobacteria-Vibrionales-Vibrionaceae-uncharacterized-uncultured_bacterium	1.65
Actinobacteria-Actinobacteria-Actinomycetales-Corynebacteriaceae-*Corynebacterium*-*Corynebacterium*_*variabile*	1.64
Proteobacteria-Alphaproteobacteria-Rhodobacterales-Rhodobacteraceae-*Roseovarius*-*Roseovarius*_sp.	1.59
Proteobacteria-Gammaproteobacteria-Alteromonadales-Alteromonadaceae-*Alishewanella*-uncultured_*Alishewanella*_sp.	1.58
Firmicutes-Clostridia-Clostridiales-Ruminococcaceae-*Faecalibacterium*-uncultured_*Faecalibacterium*_sp.	1.53
Proteobacteria-Betaproteobacteria-Burkholderiales-Burkholderiaceae-*Ralstonia*-uncultured_*Ralstonia*_sp.	1.51
Actinobacteria-Actinobacteria-Actinomycetales-uncharacterized-uncharacterized-uncultured_actinobacterium	1.47
Firmicutes-Bacilli-Lactobacillales-Streptococcaceae-*Streptococcus*-*Streptococcus*_*agalactiae*	1.46
Firmicutes-Bacilli-Bacillales-Staphylococcaceae-*Staphylococcus*-*Staphylococcus*_*pasteuri*	1.45
Firmicutes-Clostridia-Clostridiales-Lachnospiraceae-*Ruminococcus2*-uncultured_*Ruminococcus2*_sp.	1.44
Proteobacteria-Alphaproteobacteria-Sphingomonadales-Sphingomonadaceae-*Sphingorhabdus*-uncultured_*Sphingorhabdus*_sp.	1.43
Actinobacteria-Actinobacteria-Bifidobacteriales-Bifidobacteriaceae-uncharacterized-uncultured_bacterium	1.42
Proteobacteria-uncharacterized-uncharacterized-uncharacterized-uncharacterized-uncultured_bacterium	1.40
Firmicutes-Clostridia-Clostridiales-Lachnospiraceae-*Fusicatenibacter*-uncultured_*Fusicatenibacter*_sp.	1.40
Firmicutes-Clostridia-Clostridiales-Lachnospiraceae-*Blautia*-uncultured_*Blautia*_sp.	1.38
Proteobacteria-Alphaproteobacteria-Rhodobacterales-Rhodobacteraceae-*Roseovarius*-uncultured_*Roseovarius*_sp.	1.34
Firmicutes-Clostridia-Clostridiales-Clostridiales_Incertae Sedis XI-*Anaerococcus*-uncultured_*Anaerococcus*_sp.	1.31
Actinobacteria-Actinobacteria-Bifidobacteriales-Bifidobacteriaceae-*Bifidobacterium*-*Bifidobacterium*_*stellenboschense*_(T)	1.30
Firmicutes-Bacilli-Lactobacillales-Lactobacillaceae-*Lactobacillus*-*Lactobacillus*_*casei*	1.30
Actinobacteria-Actinobacteria-Bifidobacteriales-Bifidobacteriaceae-*Bifidobacterium*-*Bifidobacterium*_*breve*	1.29
Firmicutes-Erysipelotrichia-Erysipelotrichales-Erysipelotrichaceae-Erysipelotrichaceae_incertae_sedis-uncultured_Erysipelotrichaceae_sp.	1.29
Actinobacteria-Actinobacteria-Actinomycetales-Corynebacteriaceae-*Corynebacterium*-*Corynebacterium*_*flavescens*	1.28
Proteobacteria-Gammaproteobacteria-Enterobacteriales-Enterobacteriaceae-uncharacterized-*Citrobacter*_sp.	1.28
Proteobacteria-Gammaproteobacteria-Enterobacteriales-Enterobacteriaceae-*Enterobacter*-uncultured_*Enterobacter*_sp.	1.25
Proteobacteria-Gammaproteobacteria-Vibrionales-Vibrionaceae-*Vibrio*-*Vibrio*_sp.	1.25
Actinobacteria-Actinobacteria-Bifidobacteriales-Bifidobacteriaceae-*Bifidobacterium*-*Bifidobacterium*_*bifidum*	1.24
Proteobacteria-Gammaproteobacteria-uncharacterized-uncharacterized-uncharacterized-uncultured_gamma_proteobacterium	1.23
Firmicutes-Bacilli-Lactobacillales-uncharacterized-uncharacterized-*Lactobacillus*_sp.	1.23
Actinobacteria-Actinobacteria-Actinomycetales-Actinomycetaceae-*Actinomyces*-uncultured_*Actinomyces*_sp.	1.22
Proteobacteria-Gammaproteobacteria-Vibrionales-Vibrionaceae-Lucibacterium-*Vibrio*_*harveyi*	1.22
Firmicutes-Bacilli-Bacillales-Staphylococcaceae-*Staphylococcus*-uncharacterized	1.20

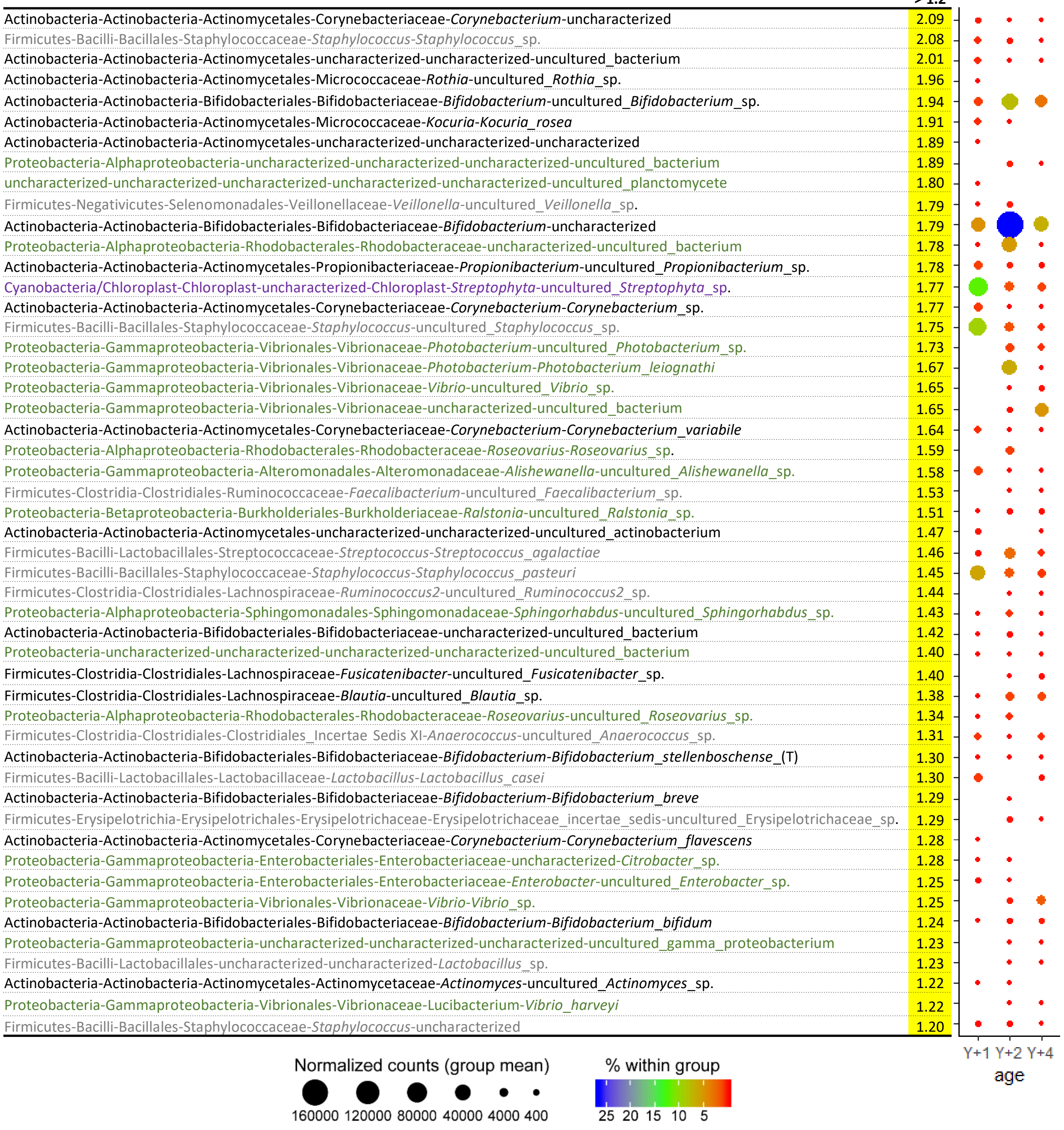

Supplementary Figure 6 | (A) Graphical representation of the variable importance (VIP) scores after component 1. (B) Dotplot map of OTUs with variable importance in projection (VIP) > 1.2 after two components. The size of the dots represents the normalized counts in each group. The color scale represents the abundance, in percentage, of each genus within each group. Y+1, Y+2 and Y+4 correspond to one-, two- and four-year-old gilthead sea bream, respectively.
