# Supplementary material for: Sex, Age, and Bacteria: How the Intestinal Microbiota Is Modulated in a Protandrous Hermaphrodite Fish
Source: Front Microbiol. 2019 Oct 31;10:2512. doi: 10.3389/fmicb.2019.02512 (PMC6834695; doi:10.3389/fmicb.2019.02512)
Supplement: Supplementary file 5 [file Table_1.DOCX]

| **Supplementary Table 1**. Detailed sequencing data. | | | | | | | | |
| --- | --- | --- | --- | --- | --- | --- | --- | --- |
| **Samples** |  | R1 | |  | R2 | |  | **Joined reads** |
|  |  | **Raw reads** | **Preprocessed reads** |  | **Raw reads** | **Preprocessed reads** |  |  |
| Y+1_2 |  | 272,602 | 244,820 |  | 272,602 | 255,684 |  | 19,028 |
| Y+1_3 |  | 286,778 | 258,158 |  | 286,778 | 269,498 |  | 12,862 |
| Y+1_4 |  | 258,108 | 234,318 |  | 258,108 | 242,162 |  | 38,401 |
| Y+1_5 |  | 279,072 | 248,726 |  | 279,072 | 260,032 |  | 21,224 |
| Y+1_6 |  | 214,676 | 192,410 |  | 214,676 | 201,160 |  | 17,708 |
| Y+1_8 |  | 355,814 | 315,664 |  | 355,814 | 327,686 |  | 15,892 |
| Y+1_9 |  | 376,424 | 332,176 |  | 376,424 | 352,464 |  | 26,548 |
| Y+1_10 |  | 303,038 | 267,836 |  | 303,038 | 278,694 |  | 21,323 |
| Y+1_11 |  | 225,836 | 202,658 |  | 225,836 | 209,722 |  | 8,484 |
| Y+1_12 |  | 169,994 | 155,096 |  | 169,994 | 155,198 |  | 5,913 |
| Y+2_1 |  | 406,908 | 364,688 |  | 406,908 | 373,596 |  | 23,661 |
| Y+2_2 |  | 274,370 | 243,366 |  | 274,370 | 255,426 |  | 21,266 |
| Y+2_3 |  | 177,496 | 160,564 |  | 177,496 | 162,596 |  | 12,023 |
| Y+2_4 |  | 308,986 | 279,712 |  | 308,986 | 281,560 |  | 12,731 |
| Y+2_5 |  | 303,178 | 274,216 |  | 303,178 | 283,950 |  | 7,652 |
| Y+2_6 |  | 301,304 | 268,906 |  | 301,304 | 281,286 |  | 116,032 |
| Y+2_7 |  | 206,628 | 185,284 |  | 206,628 | 189,266 |  | 13,595 |
| Y+2_8 |  | 217,782 | 196,360 |  | 217,782 | 199,046 |  | 7,828 |
| Y+2_9 |  | 211,626 | 189,966 |  | 211,626 | 195,080 |  | 11,739 |
| Y+2_10 |  | 323,012 | 298,650 |  | 323,012 | 303,514 |  | 102,992 |
| Y+4_1 |  | 370,876 | 325,682 |  | 370,876 | 341,678 |  | 37,439 |
| Y+4_2 |  | 298,526 | 267,850 |  | 298,526 | 278,680 |  | 11,654 |
| Y+4_3 |  | 326,226 | 291,368 |  | 326,226 | 298,486 |  | 19,879 |
| Y+4_4 |  | 225,718 | 203,074 |  | 225,718 | 206,422 |  | 8,065 |
| Y+4_6 |  | 225,470 | 202,092 |  | 225,470 | 207,834 |  | 9,534 |
| Y+4_7 |  | 263,842 | 237,890 |  | 263,842 | 246,076 |  | 15,998 |
| Y+4_8 |  | 303,492 | 274,840 |  | 303,492 | 285,482 |  | 9,461 |
| Y+4_9 |  | 392,888 | 348,442 |  | 392,888 | 361,028 |  | 18,982 |
| Y+4_10 |  | 296,552 | 269,486 |  | 296,552 | 277,404 |  | 13,473 |
| Y+4_11 |  | 372,656 | 331,948 |  | 372,656 | 347,814 |  | 25,074 |
